# Transition: Zwischen Anspruch und Versorgungsrealität: Ergebnisse aus dem InfoTrans-Projekt

**DOI:** 10.1007/s00393-026-01881-7

**Published:** 2026-10-09

**Authors:** Florian Milatz, Susanne Schalm, Kirsten Minden

**Affiliations:** 1 Forschungsbereich Epidemiologie und Versorgungsforschung, Deutsches Rheuma-Forschungszentrum Berlin (DRFZ), ein Institut der Leibniz-Gemeinschaft, Charitéplatz 1, 10117 Berlin, Deutschland; 2 Institut für Sozialmedizin, Epidemiologie und Gesundheitsökonomie, Charité – Universitätsmedizin Berlin, Gliedkörperschaft der Freien Universität Berlin und der Humboldt-Universität zu Berlin, Berlin, Deutschland; 3 Partnerstandort Berlin, Deutsches Zentrum für Kinder- und Jugendgesundheit (DZKJ), Berlin, Deutschland; 4 Rheumatologie im Zentrum, München, Deutschland; 5 Klinik für Pädiatrie mit Schwerpunkt Pneumologie, Immunologie und Intensivmedizin, Charité – Universitätsmedizin Berlin, Gliedkörperschaft der Freien Universität Berlin und der Humboldt-Universität zu Berlin, Berlin, Deutschland

**Keywords:** Juvenile idiopathische Arthritis, Versorgungsforschung, Transitionskompetenz, Patientenedukation, Routinedaten, Juvenile idiopathic arthritis, Health services research, Transition competence, Patient education, Claims data

## Abstract

**Hintergrund:**

Der Übergang von der kinder- in die internistisch-rheumatologische Versorgung stellt für junge Menschen mit juveniler idiopathischer Arthritis (JIA) eine besondere Herausforderung dar.

**Ziele:**

Untersuchung der Versorgungskontinuität, Krankheitslast, Transitionskompetenz sowie ungedeckter Bedarfe junger Erwachsener mit JIA.

**Material und Methoden:**

Im Innovationsfondsprojekt InfoTrans wurde eine Querschnittsbefragung von Barmer-Versicherten im Alter von 16–25 Jahren mit JIA durchgeführt und pseudonymisiert mit Routinedaten verknüpft. Für die vorliegende Analyse wurden ehemals kinderrheumatologisch betreute junge Erwachsene ab 20 Jahren berücksichtigt. Erfasst wurden die aktuelle Versorgungssituation, patientenberichtete Outcomes, die Transitionskompetenz (Transitions-KompAZ) sowie das psychische Wohlbefinden (WHO-5).

**Ergebnisse:**

Von den 269 befragten jungen Erwachsenen mit JIA befanden sich 45 % in fachspezifischer Betreuung. Rheumatologisch versorgte Teilnehmende wiesen eine höhere Transitionskompetenz auf (22,7 vs. 19,0; *p* < 0,001), hatten häufiger eine Übergangssprechstunde genutzt (17 % vs. 9 %) und kannten die Webseite „mein-rheuma-wird-erwachsen.de“ häufiger (33 % vs. 20 %; *p* = 0,013). Trotz vergleichbarer Krankheitslast erhielten nicht fachspezifisch Versorgte deutlich seltener krankheitsmodifizierende Antirheumatika (DMARDs; 18 % vs. 63 %). Auch bei rheumatologisch Versorgten bestanden Versorgungslücken: Zwischen der letzten kinderrheumatologischen und ersten internistisch-rheumatologischen Vorstellung lagen durchschnittlich 9,9 Monate. 35 % hatten keinen zusammenfassenden Arztbericht erhalten, mehr als 40 % waren mit der Vorbereitung auf den Betreuungswechsel unzufrieden. Insgesamt gab etwa jede bzw. jeder Fünfte relevante depressive Symptome an.

**Schlussfolgerung:**

Zwischen den Empfehlungen zur strukturierten Transition und der tatsächlichen Versorgung bestehen weiterhin relevante Diskrepanzen. Krankheitswissen scheint ein zentraler Faktor für eine kontinuierliche rheumatologische Weiterbetreuung zu sein. Die Ergebnisse unterstreichen die Bedeutung einer frühzeitigen Patientenedukation und besser integrierter Unterstützungsangebote.

## Abstract (en)

**Background:**

The transition from pediatric to adult rheumatology care represents a particular challenge for young people with juvenile idiopathic arthritis (JIA).

**Objectives:**

To investigate the continuity of care, disease burden, transition competence and unmet needs among young adults with JIA.

**Material and methods:**

Within the Innovation Fund project InfoTrans, a cross-sectional survey was conducted among- individuals insured with the Barmer health insurance aged 16–25 years with JIA and pseudonymously linked with health insurance claims data. For the present analysis, young adults aged 20 years and older with prior pediatric rheumatology care were included. Data collected comprised current healthcare utilization, patient-reported outcomes, transition competence (Transitions-KompAZ), and psychological well-being (WHO-5).

**Results:**

Among 269 surveyed young adults with JIA, 45% were receiving specialist rheumatology care. Participants receiving rheumatology care showed higher transition competence (22.7 vs. 19.0; *p* < 0.001), had more frequently attended a transition consultation (17% vs. 9%), and were more often aware of the website “mein-rheuma-wird-erwachsen.de” (33% vs. 20%; *p* = 0.013). Despite comparable disease burden, participants without specialist care received DMARD therapy less frequently (18% vs. 63%). Gaps in care were also observed among participants receiving rheumatology care: the average interval between the last pediatric rheumatology and first adult rheumatology visit was 9.9 months. Overall, 35% had not received a medical transfer summary report, more than 40% were dissatisfied with preparation for transfer, and approximately 1 in 5 participants reported relevant depressive symptoms.

**Conclusion:**

Relevant discrepancies remain between recommendations for structured transitional care and actual care delivery. Disease-related knowledge appears to be a central factor for continuous follow-up in rheumatology. The findings underline the importance of early patient education and better integration of support services.

Die juvenile idiopathische Arthritis (JIA) ist die häufigste chronisch-entzündliche rheumatische Erkrankung des Kindes- und Jugendalters. Trotz erheblicher Fortschritte in Diagnostik und Therapie besteht bei der Mehrheit der Betroffenen auch im Erwachsenenalter ein Bedarf an kontinuierlicher rheumatologischer Betreuung [16, 20]. Der Übergang von der kinder- und jugendrheumatologischen in die internistisch-rheumatologische Versorgung stellt hierbei eine klinisch und versorgungsbezogen vulnerable Phase dar [18].

Transition wird heute als strukturierter, geplanter und patientenorientierter Prozess verstanden

Transition wird heute als strukturierter, geplanter und patientenorientierter Prozess verstanden, der junge Menschen mit chronischen Erkrankungen auf die Erwachsenenmedizin vorbereitet und eine kontinuierliche Versorgung sicherstellen soll [7, 23]. Bei Jugendlichen und jungen Erwachsenen fällt dieser Übergang in eine Lebensphase, die durch zunehmende Eigenverantwortung sowie psychosoziale, schulische und berufliche Veränderungen geprägt ist [14]. Nationale und internationale Untersuchungen zeigen, dass in dieser Phase Unsicherheiten im Umgang mit der eigenen Erkrankung, Informationsverluste und Versorgungslücken auftreten können [10, 11, 13, 17, 19]. Eine unterbrochene rheumatologische Betreuung ist mit dem Risiko persistierender Krankheitsaktivität, funktioneller Einschränkungen und langfristiger Nachteile für Lebensqualität und soziale Teilhabe verbunden [13, 20].

Vor diesem Hintergrund wurden in den vergangenen Jahren Empfehlungen zur strukturierten Transition entwickelt [7, 22, 23]. In Deutschland begleitet insbesondere der gemeinsame Arbeitskreis (AK) Transition der Deutschen Gesellschaft für Rheumatologie und Klinische Immunologie (DGRh) sowie der Gesellschaft für Kinder- und Jugendrheumatologie (GKJR) seit vielen Jahren die Weiterentwicklung einer entsprechenden Versorgung. Gemeinsam mit der Deutschen Rheuma-Liga wurden verschiedene Unterlagen und Unterstützungsangebote zur Förderung einer strukturierten Transition entwickelt, darunter die peergestützte interaktive Webseite www.mein-rheuma-wird-erwachsen.de [24]. Darüber hinaus wurden weitere Unterstützungsformate wie das Transitionscamp (www.rheuma-camp.org) etabliert. Zudem bietet mittlerweile etwa die Hälfte der kinderrheumatologischen Einrichtungen strukturierte Transitionsangebote an, beispielsweise in Form gemeinsamer Übergangssprechstunden mit internistischen Rheumatolog:innen oder eines schriftlich geregelten Transitionsverfahrens [8].

Inwieweit die bestehenden Transitionsangebote die Versorgungssituation verbessert haben, wie kontinuierlich junge Menschen mit JIA fachrheumatologisch versorgt werden, welche Informations- und Unterstützungsbedarfe bestehen und welche Faktoren mit einer kontinuierlichen spezialisierten Versorgung im Erwachsenenalter assoziiert sind, wurde im Innovationsfonds-Projekt InfoTrans untersucht.

Ziel der vorliegenden Arbeit war die Analyse der Versorgungskontinuität, Krankheitslast, Transitionskompetenz sowie der Informations- und Unterstützungsbedarfe junger Erwachsener mit JIA nach Verlassen der kinderrheumatologischen Versorgung in einer auf Bevölkerungsebene über eine gesetzliche Krankenkasse rekrutierten Stichprobe.

## Methoden

### Studiendesign und -population

Das vom Innovationsfonds geförderte Projekt *InfoTrans* (Optimierung des InformationsTransfers an der Schnittstelle Kinder‑/Erwachsenenrheumatologie) wurde gemeinsam vom Deutschen Rheuma-Forschungszentrum Berlin (DRFZ), der Barmer, dem Institut für Medizinische Biometrie und Informatik der Universität Heidelberg, Patientenvertreter:innen der Deutschen Rheuma-Liga, dem Arbeitskreis Transition der rheumatologischen Fachgesellschaften, Kinderrheumatolog:innen sowie dem Berufsverband Deutscher Rheumatologen durchgeführt. Ziel des Projekts war es, den Informationstransfer zwischen der kinder- und internistisch-rheumatologischen Versorgung zu verbessern und Versorgungslücken während der Transition zu reduzieren.

Hierzu wurden zwei digitale Instrumente entwickelt und implementiert: (1) ein digitaler Patientenpass mit krankheits- und therapiebezogenen Informationen sowie Hinweisen auf Unterstützungsangebote, der Jugendlichen mit JIA über die kinderrheumatologische Dokumentationsplattform KRhOKo oder das DRFZ zur Verfügung gestellt wurde, und (2) ein digitaler Befundtransfer von der Kinderrheumatologie in die internistische Rheumatologie (d. h. zu RheDAT) über KRhOKo oder das DRFZ. Die Akzeptanz und Wirksamkeit dieser Maßnahmen wurden im Rahmen des Projekts evaluiert.

Ergänzend wurden Abrechnungsdaten sowie Daten einer Querschnittsbefragung von bei der Barmer versicherten jungen Erwachsenen im Alter von 16 bis 25 Jahren herangezogen. Die Barmer identifizierte die Studienpopulation anhand der definierten Einschlusskriterien, stellte die pseudonymisierten Routinedaten für die Analysen bereit und übernahm die Kontaktaufnahme mit den Versicherten. Die verknüpften Befragungs- und Routinedaten wurden genutzt, um die Versorgungssituation, die Zufriedenheit mit der Versorgung und ungedeckte Bedarfe während der Transition zu untersuchen.

Für die Befragung wurden Versicherte eingeschlossen, die zwischen 2018 und 2021 sowie durchgehend im Jahr 2022 bei der Barmer versichert waren und vor Vollendung des 18. Lebensjahres in mindestens zwei Quartalen Abrechnungsdiagnosen einer juvenilen Arthritis aufwiesen (ICD-10: M08 oder M09.0 für JIA, M05 oder M06 für rheumatoide Arthritis [RA], M45.X/M46.X für Spondyloarthritis [Sp] oder M07.0–M07.3, L40.5 für Psoriasisarthritis [PsA]). Die Versicherten wurden postalisch kontaktiert und konnten schriftlich oder online an der Befragung teilnehmen.

Für die vorliegende Analyse wurden ausschließlich Teilnehmende der Befragung berücksichtigt, die mindestens 20 Jahre alt waren und nach eigener Angabe vor dem Übergang in die Erwachsenenversorgung kinderrheumatologisch betreut worden waren.

### Datenerhebung

Mittels eines standardisierten Fragebogens wurden krankheitsbezogene, psychosoziale und versorgungsrelevante Parameter erhoben. Die Transitionskompetenz wurde mithilfe des validierten Instruments Transitions-KompAZ [9] erfasst. Dieses umfasst die Bereiche Krankheitswissen (0–18 Punkte), Selbstmanagement (0–15 Punkte), Inanspruchnahme und ungedeckte Bedarfe (0–21 Punkte, davon Informationsbedarf 0–10 Punkte und Bedarf an nichtmedikamentösen Maßnahmen 0–11 Punkte) sowie Zufriedenheit mit der Versorgung (0–40 Punkte, davon allgemeine Versorgung 0–20 Punkte und Transitionsprozess 0–20 Punkte).

Die subjektive Krankheitslast wurde anhand numerischer Ratingskalen (NRS 0–10) für allgemeinen Gesundheitszustand, Schmerzen, Fatigue und Einschränkungen im Alltag erfasst. Zusätzlich wurde die Anzahl schmerzhafter und geschwollener Gelenke dokumentiert. Das psychische Wohlbefinden wurde mittels WHO-5 Well-Being Index erhoben (Wertebereich 0–100; Werte ≤ 50 gelten als auffällig, Werte < 28 als Hinweis auf depressive Symptomatik) [1, 26]. Ergänzend wurden Angaben zur aktuellen Versorgungssituation, zur Inanspruchnahme transitionsbezogener Unterstützungsangebote sowie zur Kenntnis krankheitsspezifischer Informationsangebote erfasst.

Bei Teilnehmenden mit entsprechender Einwilligung erfolgte eine pseudonymisierte Verknüpfung der Befragungsdaten mit Routinedaten der Barmer. Analysiert wurden Diagnosen, Arzneimittelverordnungen sowie ambulante und stationäre Leistungsdaten. Als kontinuierliche fachrheumatologische Versorgung galt mindestens ein Kontakt zu einer internistischen Rheumatologin bzw. einem internistischen Rheumatologen innerhalb der vorangegangenen 12 Monate.

### Statistische Analyse

Die Daten wurden deskriptiv anhand von Mittelwerten, Standardabweichungen sowie absoluten und relativen Häufigkeiten beschrieben. Gruppenvergleiche zwischen kontinuierlich internistisch-rheumatologisch versorgten und nicht kontinuierlich fachrheumatologisch versorgten jungen Erwachsenen erfolgten mittels *t*-Test für kontinuierliche Variablen und χ^2^-Test für kategoriale Variablen.

Zur Identifikation von Faktoren, die mit einer kontinuierlichen internistisch-rheumatologischen Versorgung assoziiert waren, wurden univariable und multivariable logistische Regressionsanalysen durchgeführt. Potenzielle Einflussfaktoren wurden hypothesengeleitet unter Berücksichtigung klinischer Relevanz ausgewählt. Die Selektion der in das multivariable Modell eingeschlossenen Variablen erfolgte mittels Least Absolute Shrinkage and Selection Operator (LASSO) zur Berücksichtigung potenzieller Multikollinearität. Die finale Modellauswahl basierte auf dem Schwarz Bayesian Criterion.

Fehlende Werte kategorialer Variablen wurden als eigene Kategorie modelliert; für kontinuierliche Variablen erfolgte keine Imputation. Die statistischen Analysen wurden mit SAS Version 9.4 (SAS Institute, Cary, NC, USA) durchgeführt.

## Ergebnisse

### Studienpopulation

**Abb. 1:**
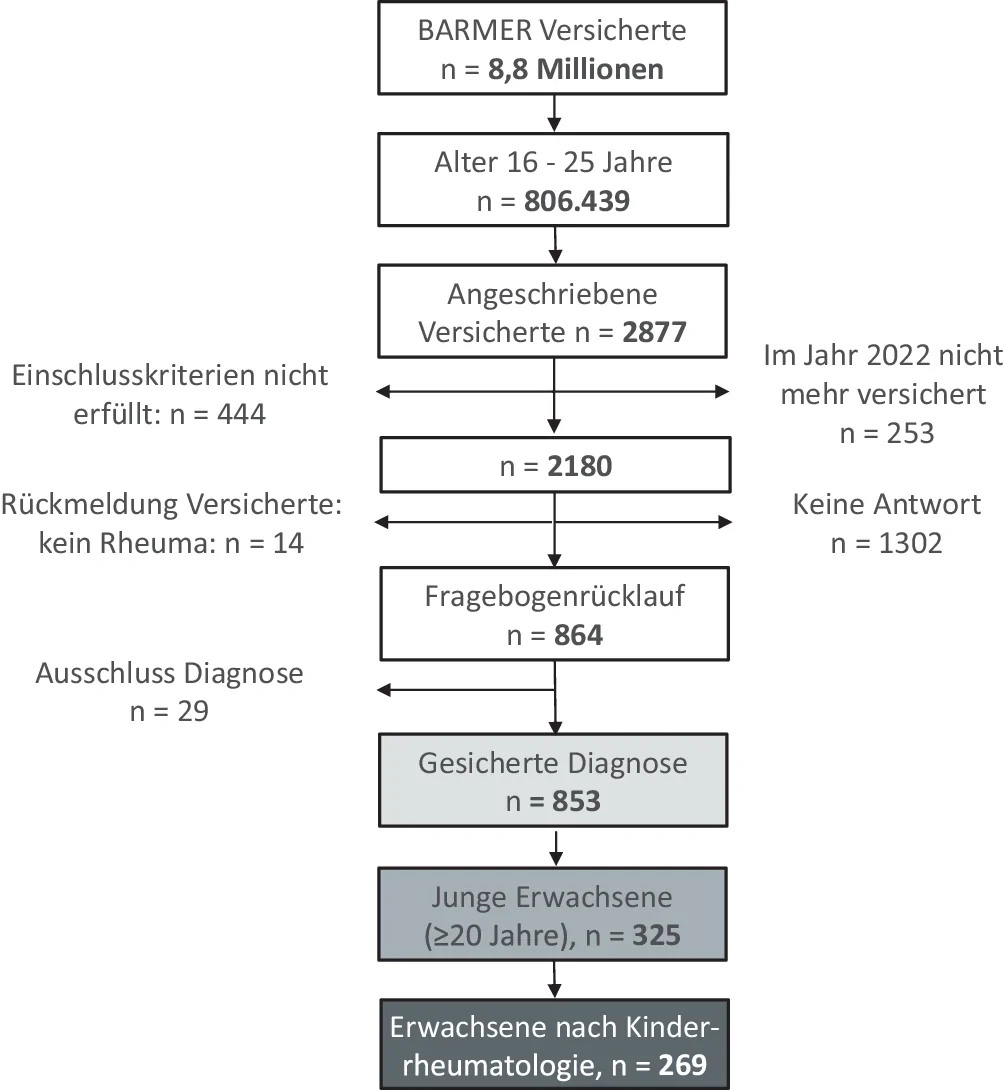
Flussdiagramm der Studienpopulation

Von 2180 angeschriebenen Barmer-Versicherten im Alter von 16 bis 25 Jahren mit JIA nahmen 853 an der Befragung teil (Response-Rate 39,1 %). Von diesen erfüllten 269 die Einschlusskriterien für die vorliegende Analyse (Alter ≥ 20 Jahre und vorangegangene kinderrheumatologische Betreuung; Abb. 1). Das mittlere Alter betrug 23,0 Jahre, 81 % der Teilnehmenden waren weiblich. Die mittlere Krankheitsdauer lag bei 11,2 Jahren (Tab. 1).

**Tab. 1:** Charakteristika vormals kinderrheumatologisch betreuter junger Erwachsener nach aktueller Versorgungssituation

Variable	Gesamt (*n* = 269)	Internistisch-rheumatologisch versorgt (*n* = 122)	Nicht fachspezifisch versorgt (*n* = 147)	*p*-Wert^1^
**Soziodemografische Merkmale**				
Alter, Jahre	23,0 (1,4)	23,0 (1,3)	23,0 (1,5)	0,669
Weibliches Geschlecht	80 %	80 %	79 %	0,774
Voll‑/Teilzeit erwerbstätig	40 %	44 %	37 %	0,107
Arbeitslos	3 %	2 %	4 %	0,237
**Krankheitslast**				
Anzahl schmerzhafter Gelenke	6,2 (9,6)	7,4 (10,2)	6,0 (9,1)	0,239
Anzahl geschwollener Gelenke	3,6 (8,1)	4,5 (9,0)	2,7 (7,1)	0,088
Gesundheitszustand (NRS 0–10)	3,2 (2,3)	3,1 (2,1)	3,3 (2,4)	0,634
Schmerzen (NRS 0–10)	2,8 (2,5)	3,0 (2,6)	2,7 (2,5)	0,297
Fatigue (NRS 0–10)	3,7 (3,2)	3,8 (3,2)	3,5 (3,2)	0,358
Einschränkung im Alltag (NRS 0–10)	2,1 (2,6)	2,3 (2,7)	2,0 (2,6)	0,293
WHO-5-Score (0–100)	51,9 (23,7)	52,5 (22,7)	51,4 (24,5)	0,717
WHO-5 Hinweis auf depressive Symptomatik (< 28)	19 %	17 %	20 %	0,114
**Inanspruchnahme medizinischer Leistungen**				
Stationäre Behandlung (letzte 12 Monate)	6,0 %	7,4 %	4,8 %	0,397
Reha-Aufenthalt (letzte 12 Monate)	0,4 %	0 %	0,7 %	0,361
Arbeitsunfähigkeit (letzte 12 Monate)	36,4 %	32,0 %	40,1 %	0,165
Anzahl AU-Tage (letzte 12 Monate)	8,9 (23,9)	5,9 (15,1)	11,4 (29,2)	0,051

Angaben als Mittelwert (Standardabweichung) bzw. Prozentangaben

*NRS* numerische Ratingskala, *WHO‑5* World Health Organization-Five Well-Being Index, *AU* Arbeitsunfähigkeit

^1^*t*-Test für unabhängige Stichproben bei metrisch verteilten Merkmalen, χ^2^-Test bei kategorialen Merkmalen

### Versorgungskontinuität und gesundheitsbezogene Outcomes

Von 269 jungen Erwachsenen hatten 122 (45 %) innerhalb der vorangegangenen 12 Monate mindestens einen Kontakt zu einer internistischen Rheumatologin bzw. einem internistischen Rheumatologen. Die mittlere Zeitspanne zwischen dem letzten kinderrheumatologischen und dem ersten internistisch-rheumatologischen Kontakt betrug 9,9 Monate (Median: 6 Monate).

Als häufigste Gründe für das Ausbleiben einer spezialisierten rheumatologischen Versorgung wurden Beschwerdefreiheit (68 %), Zugangsprobleme (19 %), fehlende Zeit (17 %), Ablehnung oder als unzureichend wirksam empfundene medikamentöse Therapie (12 %) sowie Unsicherheit oder Ängste (7 %) angegeben. Nicht fachspezifisch versorgte junge Erwachsene berichteten im Mittel über sechs schmerzhafte und drei geschwollene Gelenke. Insgesamt berichteten 86 % der jungen Erwachsenen über gesundheitliche Beeinträchtigungen, 71 % über Schmerzen.

Zwischen fachspezifisch und nicht fachspezifisch versorgten Teilnehmenden zeigten sich hierbei keine signifikanten Unterschiede hinsichtlich der subjektiv berichteten Krankheitslast (Gesundheitszustand NRS 3,1 vs. 3,3; Schmerz 3,0 vs. 2,7; Fatigue 3,8 vs. 3,5; jeweils *p* > 0,05) (Tab. 1). Internistisch-rheumatologisch versorgte junge Erwachsene erhielten jedoch häufiger krankheitsmodifizierende Antirheumatika (DMARDs; 63 % vs. 18 %; *p* < 0,001) sowie Biologika (43 % vs. 12 %; *p* = 0,015). Zudem wurden bei nicht fachspezifisch versorgten jungen Erwachsenen mehr Arbeitsunfähigkeitstage dokumentiert (Tab. 1). Insgesamt zeigten 50 % der jungen Erwachsenen ein vermindertes psychisches Wohlbefinden (WHO-5 ≤ 50), während bei 19 % Hinweise auf relevante depressive Symptome bestanden (WHO-5 < 28).

### Transitionskompetenz

**Abb. 2:**
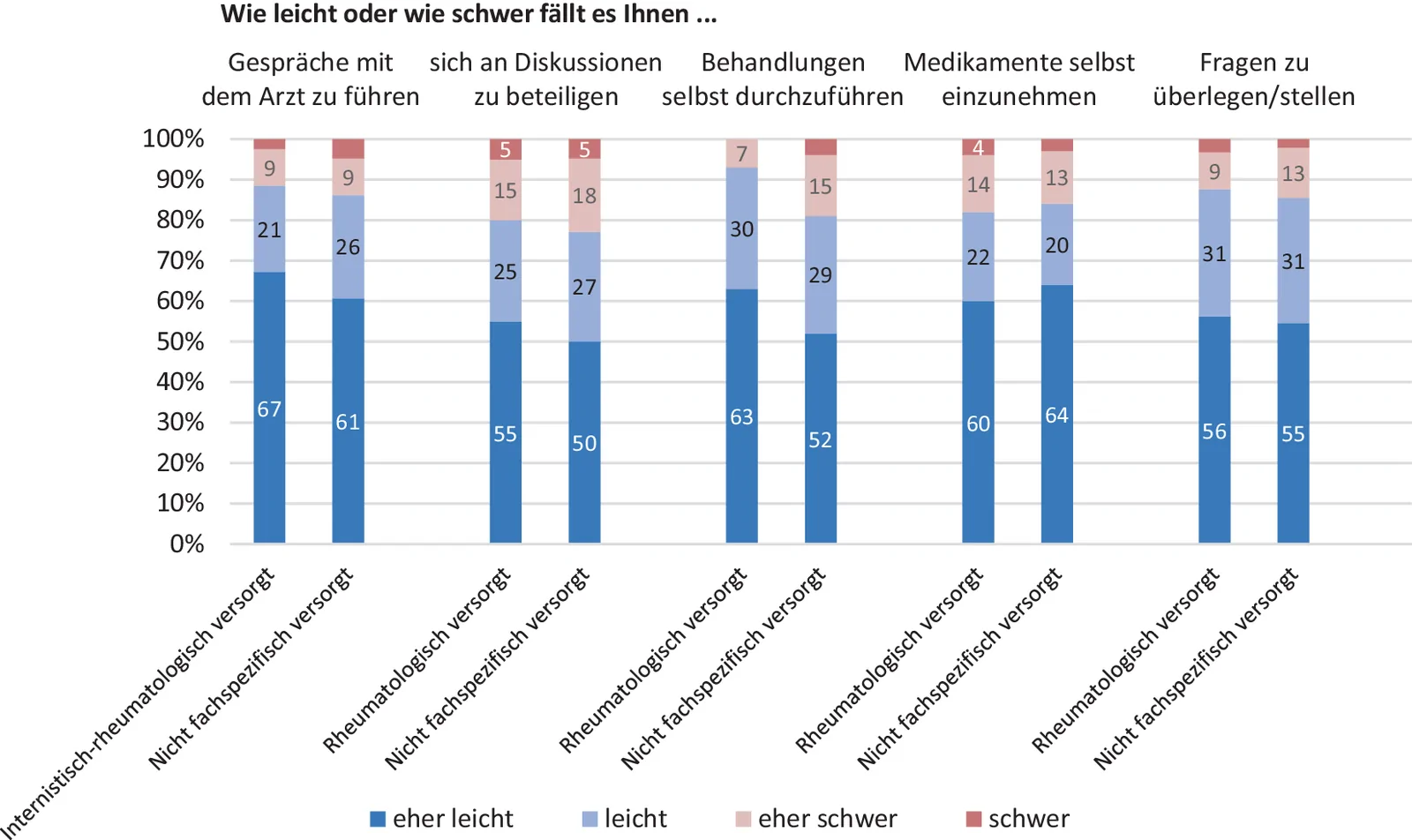
Selbstmanagementfähigkeiten junger Erwachsener nach Versorgungsart

Kontinuierlich spezialisiert versorgte junge Erwachsene wiesen eine signifikant höhere Transitionskompetenz auf als nicht spezialisiert Versorgte (Gesamtscore: 22,7 vs. 19,0; *p* < 0,001). Dies zeigte sich sowohl im Bereich des Krankheitswissens (Score: 11,4 vs. 9,1; *p* < 0,001) als auch bei den Selbstmanagementfähigkeiten (Score: 11,3 vs. 9,9; *p* = 0,002) (Tab. 2). Demnach gaben 93 % der kontinuierlich rheumatologisch Versorgten an, ihre krankheitsbezogene Behandlung selbstständig leicht oder eher leicht durchführen zu können, gegenüber 81 % der nicht fachspezifisch Versorgten. Für die eigenständige Medikamenteneinnahme zeigten sich dagegen keine relevanten Unterschiede zwischen den Gruppen (82 % vs. 84 %; Abb. 2).

**Tab. 2:** Transitionskompetenz, ungedeckte Bedarfe und Zufriedenheit bei jungen Erwachsenen, stratifiziert nach Versorgungssituation

Variable	Gesamt (*n* = 269)	Internistisch-rheumatologisch versorgt (*n* = 122)	Nicht fachspezifisch versorgt (*n* = 147)	*p*-Wert
**Transitionskompetenz**				
Transitionskompetenz gesamt (0–33)	20,7 (7,3)	22,7 (6,6)	19,0 (7,5)	**<0,001**
Krankheitswissen (0–18)	10,1 (4,7)	11,4 (4,3)	9,1 (4,9)	**<0,001**
Selbstmanagement (0–15)	10,5 (3,5)	11,3 (3,2)	9,9 (3,7)	**0,002**
**Informations- und Unterstützungsbedarf**				
Ungedeckte Bedarfe gesamt (0–21)	7,2 (4,3)	7,5 (3,9)	7,0 (4,5)	0,424
Bedarf an nichtmedikamentösen Maßnahmen (0–11)	1,9 (2,1)	2,0 (2,1)	1,8 (2,1)	0,551
Informationsbedarf (0–10)	5,3 (2,9)	5,5 (2,8)	5,1 (3,0)	0,249
**Zufriedenheit mit Versorgung**				
Zufriedenheit mit Versorgung gesamt (0–40)	23,2 (8,9)	25,2 (11,2)	20,9 (9,1)	**<0,001**
Zufriedenheit mit allgemeiner Versorgung (0–20)	13,2 (5,5)	13,8 (5,3)	13,0 (5,5)	0,193
Zufriedenheit Transitionsprozess (0–20)	10,2 (5,2)	11,3 (7,8)	7,9 (5,6)	**<0,001**
**Kenntnis transitionsbezogener Informationsangebote**				
Kenntnis der Webseite *mein-rheuma-wird-erwachsen.de*	26 %	33 %	20 %	**0,013**

Angaben als Mittelwert (Standardabweichung) bzw. Prozentangaben. Fett hervorgehobene *p*-Werte kennzeichnen statistische Signifikanz (*p* < 0,05).

**Abb. 3:**
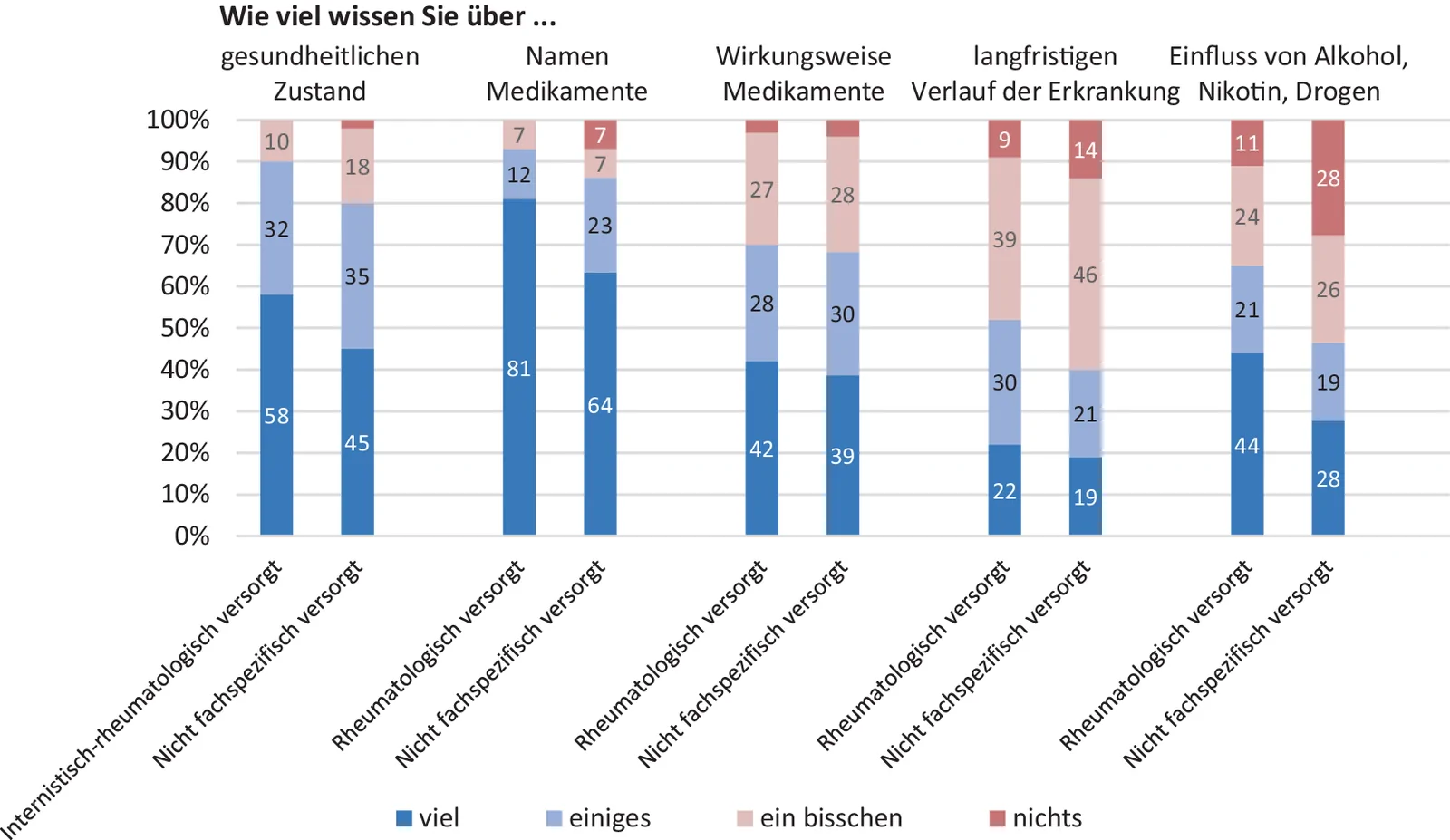
Krankheitsbezogenes Wissen junger Erwachsener nach Versorgungsart

In beiden Gruppen bestanden Wissensdefizite, insbesondere hinsichtlich Familienplanung, Substanzkonsum, langfristigem Krankheitsverlauf und Wirkmechanismen medikamentöser Therapien (Abb. 3). Kontinuierlich rheumatologisch versorgte junge Erwachsene bewerteten ihr Wissen in allen Bereichen größer als nicht fachspezifisch versorgte Teilnehmende. Gleichzeitig zeigten sich auch in dieser Gruppe Wissensdefizite: 48 % gaben geringe Kenntnisse zum langfristigen Krankheitsverlauf und 35 % zum Einfluss gesundheitsrelevanter Verhaltensweisen wie Alkohol‑, Nikotin- oder Drogenkonsum an; bei nicht fachspezifisch Versorgten lagen die entsprechenden Anteile bei 60 % bzw. 54 %.

### Inanspruchnahme und Bedarfe

Der Bedarf an nichtmedikamentösen Unterstützungsangeboten unterschied sich nicht signifikant zwischen kontinuierlich rheumatologisch versorgten und nicht fachspezifisch versorgten jungen Erwachsenen (Score: 2,0 vs. 1,8; *p* = 0,551). In der Gesamtgruppe wurden am häufigsten Bedarfe für physiotherapeutische Maßnahmen (37 %), Übergangssprechstunden (28 %) sowie strukturierte Patient:innenschulungen (27 %) angegeben. Auch unter kontinuierlich rheumatologisch versorgten jungen Erwachsenen zeigten sich relevante ungedeckte Bedarfe: So berichteten 21 % über einen Bedarf an psychologischer Beratung. Die Inanspruchnahme transitionsbezogener Angebote war insgesamt gering: Lediglich 17 % der fachspezifisch Versorgten (vs. 9 % der nicht fachspezifisch Versorgten) hatten an einer Übergangssprechstunde teilgenommen.

**Abb. 4:**
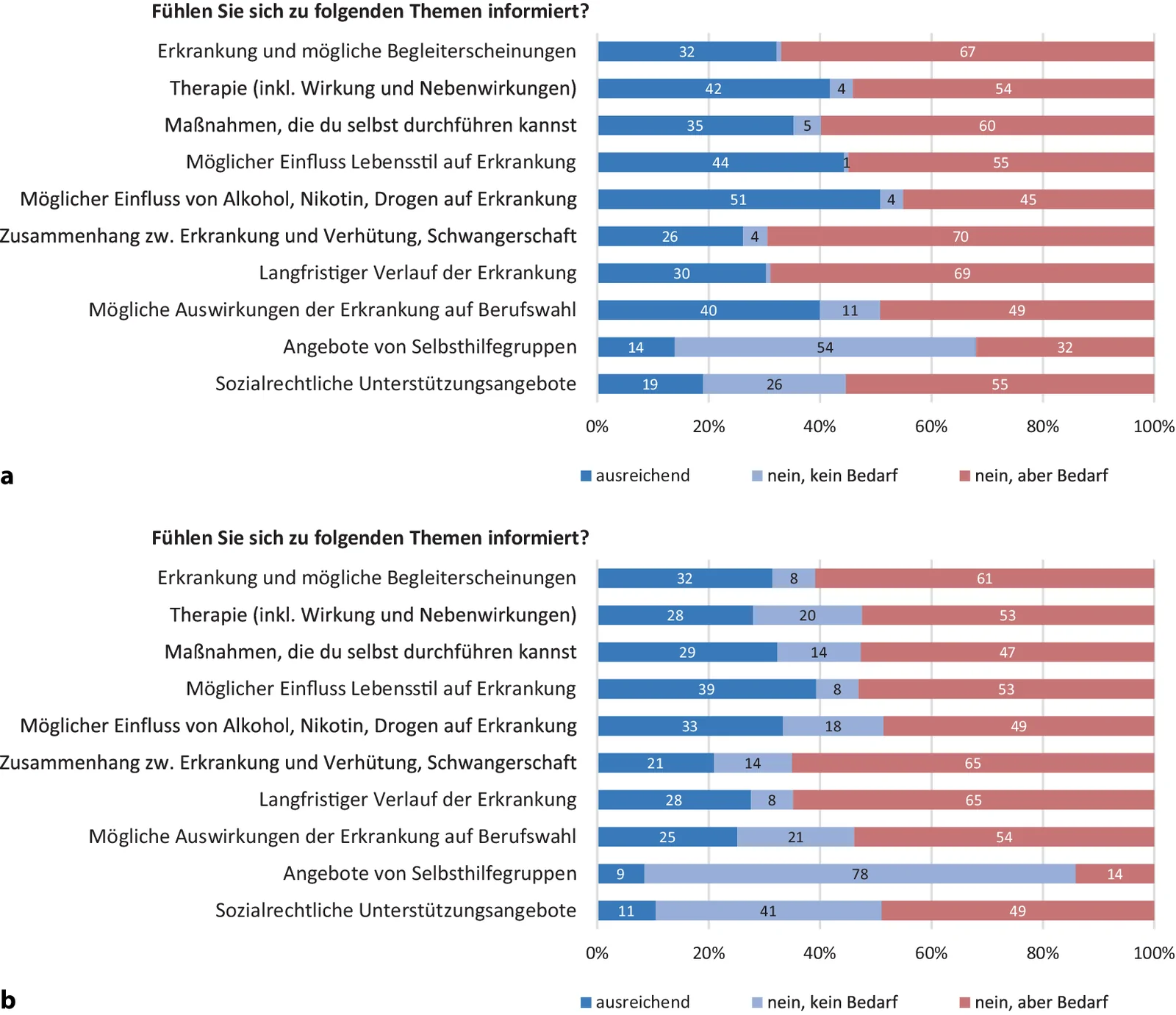
Informationsbedarfe junger Erwachsener nach aktueller Versorgungssituation. **a** Kontinuierlich internistisch-rheumatologisch versorgt (*n* = 122). **b** Nicht fachspezifisch versorgt (*n* = 147)

Der Informationsbedarf unterschied sich ebenfalls nicht signifikant zwischen beiden Gruppen (Score: 5,5 vs. 5,1; *p* = 0,249). In beiden Gruppen bestanden relevante krankheitsbezogene Informationsbedarfe, insbesondere hinsichtlich Verhütung, Schwangerschaft und Familienplanung (70 % vs. 65 %), des langfristigen Krankheitsverlaufs (69 % vs. 65 %) sowie medikamentöser Therapien und deren Nebenwirkungen (54 % vs. 53 %) (Abb. 4).

### Zufriedenheit

**Abb. 5:**
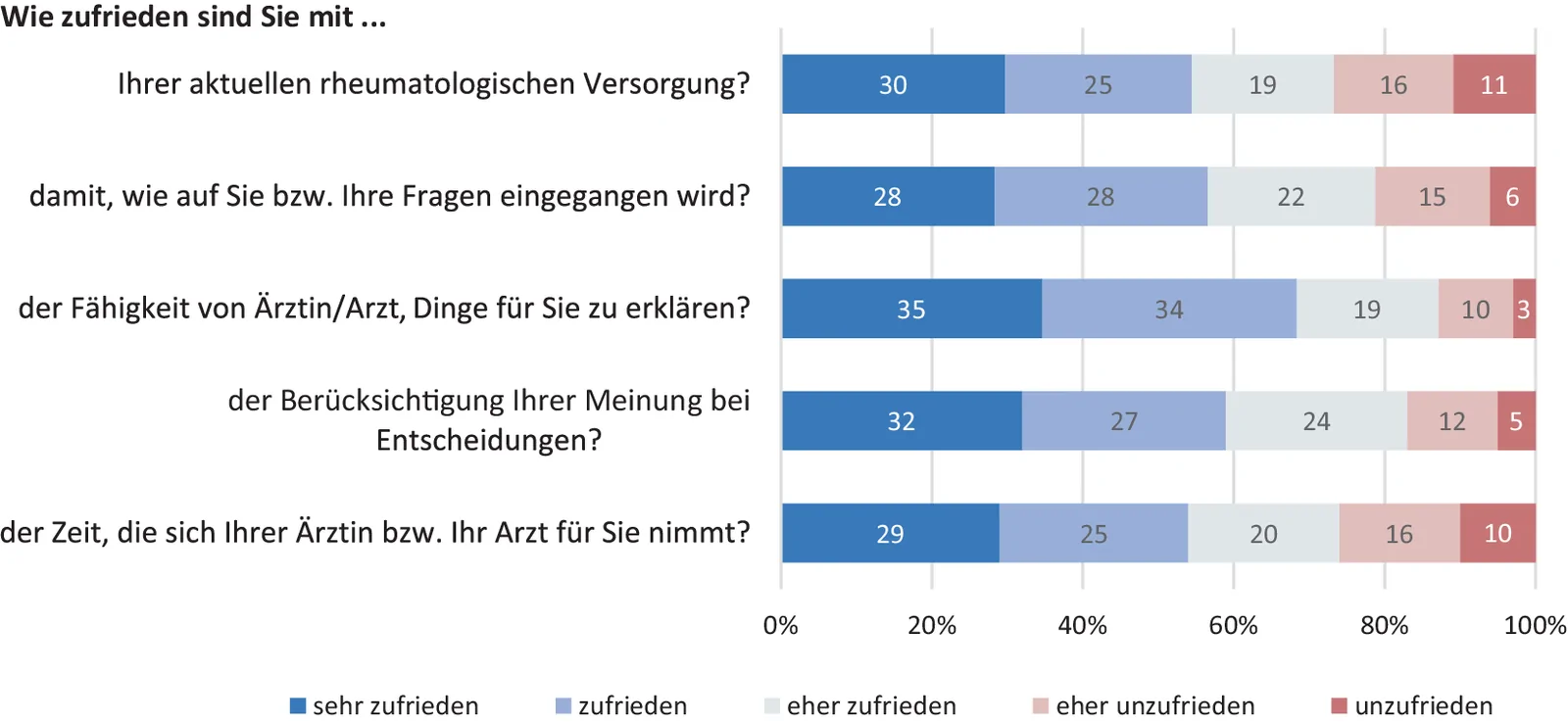
Zufriedenheit fachspezifisch versorgter junger Erwachsener mit der internistisch-rheumatologischen Versorgung

**Abb. 6:**
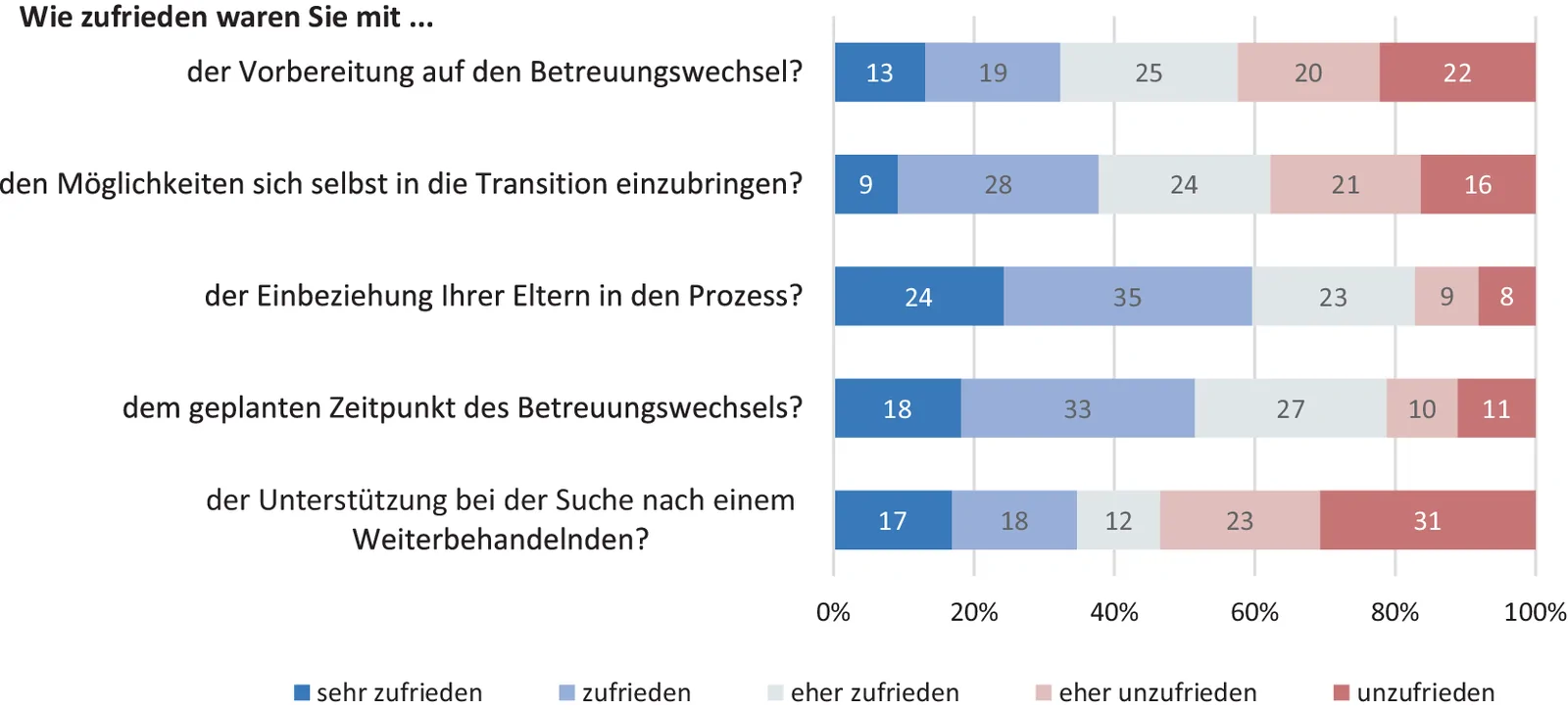
Zufriedenheit fachspezifisch versorgter junger Erwachsener mit der Transition

Internistisch-rheumatologisch versorgte junge Erwachsene berichteten über eine höhere Gesamtzufriedenheit mit der Versorgung als nicht kontinuierlich fachrheumatologisch Versorgte (Score: 25,2 vs. 20,9 Punkte; *p* < 0,001). Signifikante Gruppenunterschiede zeigten sich im transitionsspezifischen Zufriedenheitsscore (Tab. 2). Unter kontinuierlich internistisch-rheumatologisch versorgten jungen Erwachsenen wurde die allgemeine Versorgung insgesamt überwiegend positiv bewertet (Abb. 5). Bezogen auf einzelne Aspekte des Transitionsprozesses zeigten sich hingegen relevante Unzufriedenheiten: 42 % waren mit der Vorbereitung auf den Betreuungswechsel unzufrieden, 37 % zeigten sich mit ihren Möglichkeiten zur aktiven Einbringung in den Transitionsprozess unzufrieden, und 54 % gaben an, keine ausreichende Unterstützung bei der Suche nach einer internistischen Rheumatologin bzw. einem internistischen Rheumatologen erhalten zu haben (Abb. 6). Darüber hinaus berichteten 35 %, keinen zusammenfassenden ärztlichen Übergabebrief erhalten zu haben. 17 % verfügten über den vom AK Transition herausgegebenen Transitionspass, und 26 % hatten aufgrund von Unzufriedenheit bereits die internistische Rheumatologin bzw. den internistischen Rheumatologen gewechselt.

### Kenntnis transitionsbezogener Informationsangebote

Insgesamt kannten 23 % der Befragten die Webseite *mein-rheuma-wird-erwachsen.de*. Spezialisiert versorgte junge Erwachsene gaben deutlich häufiger an, die Webseite zu kennen als nicht spezialisiert versorgte Teilnehmende (33 % vs. 20 %; *p* = 0,013). Die Informationsangebote der Fachgesellschaften waren hingegen lediglich 5,2 % der Befragten bekannt.

### Faktoren assoziiert mit kontinuierlicher spezialisierter Versorgung

In univariablen Analysen waren höhere Transitionskompetenz, d. h. stärker ausgeprägtes Krankheitswissen und bessere Selbstmanagementfähigkeiten, sowie die Kenntnis der Webseite *mein-rheuma-wird-erwachsen.de* mit einer kontinuierlichen internistisch-rheumatologischen Versorgung assoziiert. Im multivariablen Regressionsmodell blieb höheres Krankheitswissen als einziger unabhängiger Faktor signifikant assoziiert (OR 1,07; 95 % KI 1,01–1,15; *p* = 0,034) (Tab. 3).

**Tab. 3:** Faktoren assoziiert mit kontinuierlicher internistisch-rheumatologischer Versorgung

Variable	Univariable Analyse			Multivariable Analyse*		
	OR	95 % KI	*p*-Wert	OR	95 % KI	*p*-Wert
**Transitionskompetenz**						
Transitionskompetenz, Gesamtscore (0–33)	1,07	1,04–1,11	< 0,001	–	–	–
Krankheitswissen (0–18)	1,11	1,05–1,18	< 0,001	1,07	1,01–1,15	**0,034**
Selbstmanagementfähigkeiten (0–15)	1,12	1,04–1,21	0,002	1,06	0,97–1,16	0,213
**Informations- und Unterstützungsbedarf**						
Ungedeckte Bedarfe, Gesamtscore (0–21)	1,02	0,97–1,09	0,428	–	–	–
Bedarf an nichtmedikamentösen Maßnahmen (0–11)	1,04	0,92–1,17	0,510	–	–	–
Informationsbedarf (0–10)	1,05	0,97–1,14	0,252	–	–	–
**Zufriedenheit mit Versorgung**						
Zufriedenheit mit Versorgung gesamt (0–40)	1,01	0,97–1,05	0,771	–	–	–
Zufriedenheit mit allgemeiner Versorgung (0–20)	1,04	0,98–1,11	0,174	–	–	–
Zufriedenheit Transitionsprozess (0–20)	0,97	0,91–1,04	0,393	–	–	–
**Weitere Faktoren**						
Weibliches Geschlecht	1,09	0,60–1,98	0,775	–	–	–
Vermindertes Wohlbefinden (WHO-5 ≤ 50)	0,83	0,44–1,54	0,546	–	–	–
Kenntnis der Webseite *mein-rheuma-wird-erwachsen.de*	2,03	1,16–3,55	0,013	1,57	0,87–2,84	0,135

Variablenselektion für das multivariable Modell erfolgte mittels LASSO-Regression. Fett hervorgehobene *p*-Werte kennzeichnen statistische Signifikanz (*p* < 0,05).

## Diskussion

Die Ergebnisse der vorliegenden Befragung auf Bevölkerungsebene zeigen, dass zwischen bestehenden Empfehlungen zur strukturierten Transition und der realen Versorgungssituation junger Erwachsener mit JIA weiterhin deutliche Diskrepanzen bestehen. Weniger als die Hälfte der vormals kinderrheumatologisch betreuten jungen Erwachsenen befand sich zum Befragungszeitpunkt in kontinuierlicher internistisch-rheumatologischer Betreuung. Gleichzeitig wurden auch unter nicht fachspezifisch versorgten Teilnehmenden weiterhin gesundheitliche Beeinträchtigungen berichtet.

Im Vergleich zu internationalen [6] und nationalen [17] Ergebnissen, fallen die beobachteten Versorgungsraten damit geringer aus und sprechen deutlich für fortbestehende Versorgungslücken im Übergang in die Erwachsenenmedizin.

Auch die beobachtete mittlere Zeitspanne zwischen der letzten kinderrheumatologischen und der ersten internistisch-rheumatologischen Vorstellung spricht für relevante Unterbrechungen der spezialisierten Versorgung. Mit durchschnittlich knapp 10 Monaten überschreitet sie deutlich den von Kinderrheumatolog:innen als akzeptabel angesehenen Zeitraum von maximal 6 Monaten [4]. Vergleichbare Verzögerungen wurden auch in internationalen Untersuchungen beschrieben [21] und gelten als klinisch relevant, da sie mit Therapieabbrüchen, reduzierter Adhärenz und einem erhöhten Risiko ungünstiger Krankheitsverläufe assoziiert sein können [5].

Strukturierte Unterstützungsangebote erreichen nur einen begrenzten Teil der Zielgruppe

Ein zentrales Ergebnis der vorliegenden Untersuchung ist zudem die Beobachtung, dass bestehende strukturierte Unterstützungsangebote bislang nur einen begrenzten Teil der Zielgruppe erreichen. Nur ein kleiner Anteil der Befragten berichtete von der Teilnahme an Übergangssprechstunden oder verfügte über etablierte Instrumente wie einen Transitionspass oder einen strukturierten ärztlichen Übergabebrief. Zusätzlich zeigte sich eine geringe Nutzung und Bekanntheit etablierter Informations- und Unterstützungsangebote innerhalb der Zielgruppe. Bemerkenswert ist zudem, dass auch bei kontinuierlich internistisch-rheumatologisch versorgten jungen Erwachsenen weiterhin relevante Informations- und Unterstützungsbedarfe bestanden.

Diese Befunde sprechen dafür, dass trotz bestehender nationaler Initiativen weiterhin relevante Defizite an der Schnittstelle zwischen pädiatrischer und internistisch-rheumatologischer Versorgung bestehen. Strukturelle Versorgungsbarrieren, darunter der seit Jahren bestehende Mangel internistisch-rheumatologischer Versorgungskapazitäten sowie regionale Unterschiede in der Verfügbarkeit spezialisierter Angebote, erschweren die Kontinuität der Betreuung zusätzlich [2, 3].

Bemerkenswert erscheint darüber hinaus die Diskrepanz zwischen subjektiv angegebener Beschwerdefreiheit als häufigstem Grund für das Ausbleiben einer spezialisierten Weiterbetreuung und gleichzeitig fortbestehender Krankheitslast innerhalb der Gruppe ohne kontinuierliche fachrheumatologische Versorgung. Obwohl viele Betroffene fehlende Beschwerden als Ursache für die ausbleibende Weiterbetreuung nannten, berichtete ein relevanter Anteil weiterhin über Schmerzen, geschwollene Gelenke und gesundheitliche Einschränkungen. Dieses Ergebnis legt nahe, dass eine subjektiv als stabil wahrgenommene Erkrankung nicht zwangsläufig mit einer tatsächlich niedrigen Symptomlast einhergeht und Defizite im Krankheitsverständnis sowie im gesundheitsbezogenen Selbstmanagement den weiteren Versorgungspfad beeinflussen könnten.

Die vorliegenden Ergebnisse deuten darauf hin, dass individuelle krankheitsbezogene Kompetenzen eine relevante Rolle für die Versorgungskontinuität im Transitionsprozess spielen. In der multivariablen Analyse erwies sich Krankheitswissen als unabhängiger Faktor, der signifikant mit einer kontinuierlichen internistisch-rheumatologischen Versorgung assoziiert war. Gleichzeitig zeigten sich in mehreren krankheitsbezogenen Themenbereichen weiterhin relevante Wissensdefizite und Informationsbedarfe, u. a. hinsichtlich des langfristigen Krankheitsverlaufs, therapeutischer Maßnahmen, Verhütung und Schwangerschaft sowie gesundheitsrelevanter Lebensstilfaktoren. Diese Befunde unterstreichen die Bedeutung frühzeitiger und strukturierter Patientenedukation bereits im Kindes- und frühen Jugendalter und sprechen dafür, die Förderung von Krankheitsverständnis und Eigenverantwortung stärker in bestehende Transitionskonzepte zu integrieren [12, 14].

Die vorliegenden Ergebnisse legen zudem nahe, dass zukünftige Interventionen nicht nur auf die Bereitstellung bestehender Informations- und Unterstützungsangebote abzielen, sondern deren aktive Nutzung und Integration in den Transitionsprozess fördern sollten. Erfolgversprechende Ansätze könnten die stärkere Einbindung digitaler, niedrigschwelliger und bedarfsorientierter Unterstützungsangebote sowie verbindlichere Übergabestrukturen mit aktiver Anbindung an die Erwachsenenrheumatologie umfassen.

Psychische Belastungen stellen einen potenziell relevanten Faktor im Transitionsprozess dar

Neben strukturellen und edukativen Herausforderungen zeigte sich darüber hinaus eine hohe psychosoziale Belastung der jungen Erwachsenen. Etwa jede zweite befragte Person zeigte Hinweise auf ein eingeschränktes psychisches Wohlbefinden, jede fünfte Person Hinweise auf eine relevante depressive Symptomatik. Eine vergleichbar hohe Prävalenz psychischer Belastungen wurde auch in aktuellen Untersuchungen bei Jugendlichen mit JIA beschrieben [15]. Psychische Belastungen können die Krankheitsbewältigung, das gesundheitsbezogene Selbstmanagement sowie die kontinuierliche Inanspruchnahme medizinischer Versorgung beeinflussen und stellen damit einen potenziell relevanten Faktor im Transitionsprozess dar [25]. Gleichzeitig zeigte sich ein relevanter Bedarf an psychologischer Unterstützung, dem in der Versorgungspraxis bislang offenbar nur unzureichend begegnet wird. Vor dem Hintergrund begrenzter psychotherapeutischer Versorgungskapazitäten erscheinen ein routinemäßiges psychosoziales Screening im Transitionsprozess, die frühzeitige Identifikation besonders belasteter junger Menschen sowie die bedarfsgerechte Vermittlung an geeignete psychosoziale Beratungs- und Unterstützungsangebote als sinnvolle Maßnahmen zur Verbesserung der Versorgung.

Zu den Stärken der Studie zählen die Rekrutierung auf Bevölkerungsebene über eine große gesetzliche Krankenkasse, die Verknüpfung von Befragungs- und Routinedaten sowie der Einsatz validierter Instrumente zur Erfassung von Transitionskompetenz und patientenberichteten Outcomes. Einschränkend sind das querschnittliche Studiendesign, potenzielle Erinnerungsverzerrungen bei retrospektiven Angaben zum Transitionsprozess, die routinedatenbasierte Definition einer kontinuierlichen spezialisierten Versorgung ohne Informationen zu Inhalt und Frequenz der Betreuung sowie das Fehlen objektiver klinischer Krankheitsaktivitätsparameter zu berücksichtigen. Obwohl Teilnehmende und Nichtteilnehmende hinsichtlich Alter und Diagnoseverteilung anhand der verfügbaren Routinedaten vergleichbar waren, können Selektionsprozesse infolge der Rücklaufquote nicht vollständig ausgeschlossen werden.

Insgesamt zeigen die Ergebnisse, dass der Übergang junger Menschen mit JIA in die Erwachsenenversorgung weiterhin eine vulnerable Phase mit relevanten Versorgungslücken darstellt. Künftige Studien sollten insbesondere evaluieren, welche strukturellen und edukativen Interventionen langfristig geeignet sind, Versorgungskontinuität und patientenrelevante Outcomes nachhaltig zu verbessern.

## Fazit für die Praxis

- Ein relevanter Anteil junger Erwachsener mit juveniler idiopathischer Arthritis (JIA) wird nach Verlassen der Kinderrheumatologie nicht kontinuierlich fachspezifisch weiterbetreut.
- Eine fehlende spezialisierte Versorgung geht mit einer deutlich geringeren DMARD-Anwendung einher.
- Ein höheres Krankheitswissen ist mit einer besseren Versorgungskontinuität assoziiert und sollte bereits im Jugendalter gezielt gefördert werden.
- Bestehende Unterstützungsangebote zur Transition sollten jungen Menschen mit JIA in der Routineversorgung aktiv vermittelt und niedrigschwellig zugänglich gemacht werden.
- Psychosoziale Belastungen (z. B. depressive Symptome) sind häufig und sollten im Transitionsprozess regelhaft erfasst und berücksichtigt werden.
- Strukturierte Übergabedokumentation und klare Weiterempfehlungen können die Versorgungskontinuität verbessern.

## Data Availability

Die in dieser Studie erhobenen Datensätze können auf begründete Anfrage beim Korrespondenzautor angefordert werden.
